# Transfer hospitalizations for pediatric severe sepsis or septic shock: resource use and outcomes

**DOI:** 10.1186/s12887-019-1577-5

**Published:** 2019-06-13

**Authors:** Folafoluwa O. Odetola, Achamyeleh Gebremariam

**Affiliations:** 1 Department of Pediatrics and Communicable Diseases, Division of Pediatric Critical Care Medicine, 6C07, 300 North Ingalls Street, Ann Arbor, MI 48109 USA; 2 Child Health Evaluation and Research Center, University of Michigan, Ann Arbor, MI 48109 USA

**Keywords:** Sepsis, Hospitalized children, Mortality, Teaching hospitals, Length of stay, Hospital charges

## Abstract

**Background:**

Sepsis is a major cause of child mortality and morbidity. To enhance outcomes, children with severe sepsis or septic shock often require escalated care for organ support, sometimes necessitating interhospital transfer. The association between transfer admission for the care of pediatric severe sepsis or septic shock and in-hospital patient survival and resource use is poorly understood.

**Methods:**

Retrospective study of children 0–20 years old hospitalized for severe sepsis or septic shock, using the 2012 Kids’ Inpatient Database. After descriptive and bivariate analysis, multivariate regression methods assessed the independent relationship between transfer status and outcomes of in-hospital mortality, duration of hospitalization, and hospital charges, after adjustment for potential confounders including illness severity.

**Results:**

Of an estimated 11,922 hospitalizations (with transfer information) for pediatric severe sepsis and septic shock nationally in 2012, 25% were transferred, most often to urban teaching hospitals. Compared to non-transferred children, transferred children were younger, and had a higher frequency of extreme illness severity (84% vs. 75%, *p* < .01), and of multiple organ dysfunction (32% vs. 24%, p < .01). They also had higher use of invasive medical devices including arterial catheters, invasive mechanical ventilation, and central venous catheters; and of specialized technology, including renal replacement therapy (6.2% vs. 4.6%, *p* < .01) and extracorporeal membrane oxygenation (5.7% vs. 1.8%, p < .01). Transferred children had longer hospitalization and accrued higher charges than non-transferred children (p < .01). Crude mortality was higher among transferred than non-transferred children (21.4% vs.15.0%, p < .01), a difference no longer statistically significant after multivariate adjustment for potential confounders (Odds Ratio:1.04, 95% Confidence interval: 0.88–1.24). Similarly, adjusted length of hospital stay and hospital charges were not statistically different by transfer status.

**Conclusion:**

One in four children with severe sepsis or septic shock required interhospital transfer for specialized care associated with greater use of invasive medical devices and specialized technology. Despite higher crude mortality and resource consumption among transferred children, adjusted mortality and resource use did not differ by transfer status. Further research should identify quality-of-care factors at the receiving hospitals that influence clinical outcomes and resource use.

## Background

Sepsis is a major contributor to child mortality and morbidity [1–3], with significant in-hospital cost burden [1, 3]. The occurrence of organ dysfunction in the septic state, exemplified by severe sepsis or septic shock, is associated with elevated risk of adverse outcomes including death [4]. To ameliorate organ dysfunction, patients with severe sepsis and septic shock often require escalated care to provide artificial organ support such as continuous renal replacement therapy (CRRT) or extracorporeal membrane oxygenation (ECMO). Given the differential capacity of hospitals to provide such specialized resources, deployment of these advanced organ-supportive therapeutic modalities often requires transfer to hospitals with such resources [5, 6].

Given the time-sensitive nature of care for children with severe sepsis or septic shock, and the detrimental consequences of delayed definitive and resuscitative care, including lower survival and increased morbidity [7, 8], it is important to investigate for any association between transfer admission for the care of pediatric severe sepsis or septic shock and in-hospital patient survival and resource use. The research might elucidate opportunities to alleviate illness burden and the associated resource use burden and provide insight to approaches to improve healthcare delivery for children with sepsis.

The study was conducted to test the hypothesis that children with severe sepsis or septic shock who undergo interhospital transfer have higher in-hospital mortality, longer hospitalization, and higher overall hospital charges compared with those not transferred.

Some of the results of this study have been previously reported in the form of an abstract [9].

## Methods

### Study design

We conducted a retrospective study of hospitalized children 0–20 years old, with severe sepsis or septic shock. Our data source was the 2012 Kids’ Inpatient Database (KID), Healthcare Cost and Utilization Project (HCUP), developed by the Agency for Healthcare Research and Quality. The KID sample includes approximately 3 million pediatric discharge records obtained from 4179 hospitals in 44 states, drawn from the four major U.S. census regions [10]. The KID is the only national, all-payer database of hospitalizations for children. The database is publicly available and contains 80% of the normal non-newborn discharges from these states and is nationally representative with the inclusion of discharge weights in analyses. Information on patient demographics, hospital characteristics, and diagnosis/procedure codes is included for each hospitalization [10].

### Study sample and variable identification

International Classification of Diseases, ninth revision, clinical modification (ICD-9-CM) codes were used to identify children with a primary or secondary diagnosis of severe sepsis (995.92) or septic shock (785.52).

Dependent (outcome) variables: In-hospital mortality, hospital length of stay (LOS), and hospital charges.

Independent variables:

- The exposure variable for the analysis was the patient transfer status (transfer hospitalizations to the study hospital vs. non- transfer hospitalizations).
- Patient characteristics of study interest were: age (categorized: 0–11 months; and 1–5, 6–10, 11–15, 16–20 years), sex, number of dysfunctional organ systems, presence and count of comorbidities, primary insurance payer (categorized: public, private, and self-pay/other), and severity of illness using the All-patient refined diagnosis related group (APRDRG) classification within the KID [10]. The categories of APRDRG-severity of illness – minor, moderate, major, and extreme – are hereafter referred to as illness severity. The APRDRG severity classification is a proprietary, validated, and extensively used measure of illness severity that utilizes patient discharge data including principal and secondary diagnoses, procedures, and demographic information to assign patients to subclasses of illness severity [10]. Due to small sample size for hospitalizations with minor illness severity, the categories for minor and moderate illness severity were combined for the analyses. Applying methodology previously described in the literature [11], Comorbidities were identified using ICD-9-CM codes, and displayed in Appendix A. Organ dysfunction was similarly identified using ICD-9-CM codes, applying methodology previously described in the literature [12], and displayed in Appendix B.
- Hospital characteristics of study interest were: the type of hospital (categorized: location and teaching status: teaching urban, non-teaching urban, and rural hospital), and the census region [10]. Teaching hospital status was determined by whether a hospital had an American Medical Association-approved residency program, was a member of the Council of Teaching Hospitals, or had a ratio of full-time equivalent interns and residents to beds of .25 or higher [10]. Rural-urban designation of hospitals was determined by the core-based statistical area (CBSA). Hospitals residing in counties with a CBSA type of metropolitan were considered urban, while hospitals with a CBSA type of micropolitan or non-core were classified as rural [10]. Due to the rarity of rural teaching hospitals, data from rural hospitals were not specified by teaching status in the KID [10]. Of note, for children who underwent interhospital transfer, the study hospital is the receiving hospital.
- Use of invasive medical technology during hospitalization was determined using ICD-9-CM procedure codes. These included:
  I. Invasive medical devices: invasive mechanical ventilation (96.7), arterial catheterization (38.91), tracheostomy (31.1–31.29), central venous catheterization (38.93, 89.62); and,
  II. Specialized medical technology: continuous renal replacement therapy/dialysis – CRRT (38.95, V45.11, 39.95), and extracorporeal membrane oxygenation – ECMO (39.65).
  III. Cardiopulmonary resuscitation – CPR (99.60). This procedure was included as an invasive procedure given prior report of elevated risk of mortality if CPR occurred around the time of interhospital transfer of children to intensive care settings [13].

The Institutional Review Board of the University of Michigan Medical School approved the study.

### Statistical analysis

The number of hospitalizations for severe sepsis or septic shock was determined after excluding hospitalizations with missing transfer information. Thereafter, patient and hospital characteristics, and the frequency of use of invasive medical technology were compared according to the transfer status of the patients. Additionally, to assess for potential confounding, the outcome variables of in-hospital mortality, hospital LOS, and overall hospital charges were compared among hospital and patient characteristics, invasive medical technology, and patient transfer status. Independent variables associated with the outcome variables with *p* value ≤0.20 in bivariate analyses, and those that statistically differed by transfer status with p value ≤0.20, were included in multivariate regression models which were fit to assess the independent association between transfer status and the outcome variables. In these analyses, multivariable logistic regression, negative binomial regression and multiple linear regression models for complex survey data were fit to assess the independent association of transfer status with in-hospital mortality, LOS, and hospital charges respectively, after adjustment for potential confounders including illness severity.

The number of hospitalizations in the results was unweighted, while all effect estimates and accompanying 95% confidence intervals were calculated using sample weights to account for the complex survey design and obtain national estimates. All estimates used the survey commands in Stata for Windows (Stata Corp.; College Station, Texas) version 15, which accounted for the complex survey design. To allow generation of stable estimates, cell frequencies that were too small (< 70) for precise estimation were suppressed in the report as recommended by AHRQ [10].

## Results

There were 8533 hospitalizations with severe sepsis or septic shock with transfer information in the database, representing an estimated 11,922 hospitalizations for pediatric severe sepsis or septic shock nationally in 2012. Of the 8533 hospitalizations, 2151 (25.4%) were associated with interhospital transfer, most often to urban teaching hospitals (Table 1). In the overall study cohort, extreme illness severity was observed in 77% of hospitalizations, comorbidities in 75% of children, and multiple (2 or more organ-system involvement) organ dysfunction in 26.4%. In-hospital mortality was 17%, average length of stay was 22 days, and the average charge per hospitalization was $314,950.

**Table 1:** Distribution of patient and hospital characteristics according to transfer status

Characteristic	Overall Hospitalizations (*n* = 8533) %	Transfer Hospitalizations (*n* = 2151) %	Non-transfer Hospitalizations (*n* = 6382) %	p
Age in Years				
< 1	29.1	39.6	25.5	
1–5	15.1	14.0	15.5	
6–10	9.0	8.1	9.2	
11–15	13.6	14.1	13.5	
16–20	33.2	24.2	36.3	< 0.0001
Female Sex	48.9	47.3	49.5	0.07
APRDRG Levels of Illness Severity				
Minor/Moderate	5.1	3.3	5.8	
Major	17.8	12.5	19.7	
Extreme	77.0	84.2	74.5	< 0.0001
Primary Payer				
Public	51.9	55.6	50.6	
Private	37.4	33.5	38.8	
Self-pay/Other	10.7	10.9	10.6	0.0015
Type of Hospital				
Rural	2.1	1.2	2.4	
Urban Non-Teaching	12.7	4.6	15.4	
Urban Teaching	85.2	94.2	82.2	< 0.0001

In bivariate analysis, patient and hospital characteristics varied by transfer status. In comparison with non-transferred children, transferred children were younger and had higher frequency of extreme illness severity (84% vs. 75%, *p* < .01) (Table 1). Multiple organ dysfunction also occurred in higher frequency among transferred than non-transferred children (32% vs. 24%, *p* < .01). There was no difference in the frequency of comorbidities by transfer status.

Transfer hospitalization was notably associated with higher use of invasive medical devices including arterial catheters, invasive mechanical ventilation, central venous catheters; and specialized technology, including CRRT and ECMO (Table 2). The occurrence of CPR did not differ by transfer status, occurring in 3.8% of transferred and 3.9% of non-transferred children (*p* = 0.84).

**Table 2:** Distribution of invasive medical technology according to transfer status

Characteristic	Overall Hospitalizations (*n* = 8533) %	Transfer Hospitalizations (*n* = 2151) %	Non-transfer Hospitalizations (*n* = 6382) %	p
Invasive Medical Technology				
Mechanical ventilation	51.4	65.4	46.6	< 0.0001
Arterial catheterization	23.1	27.0	21.8	< 0.0001
Central venous catheterization	37.4	40.9	36.2	0.0006
Tracheostomy	3.8	5.5	3.3	< 0.0001
ECMO	2.8	5.7	1.8	< 0.0001
CRRT	5.0	6.2	4.6	0.0072

Unadjusted outcomes and resource use differed significantly between transferred and non-transferred children. Transferred children had longer hospitalization (30 vs. 22 days, *p* < .05) and accrued higher hospital charges ($385,275 vs. $290,882, p < .05). Crude mortality was also higher among transferred than non-transferred children (21.4% vs.15.0%, *p* < .01).

After multivariate adjustment for potential confounders, adjusted in-hospital mortality was not statistically different between transferred and non-transferred children (Odds Ratio:1.04, 95% Confidence interval [CI]:0.88–1.24). Similarly, adjusted length of hospital stay (1.1 days; 95% CI: − 1.2-3.4 days) and hospital charges (− 2814; 95% CI: − 31,656 – 26,028) for transferred versus non-transferred children were not statistically different.

## Discussion

One out of every four hospitalizations for children with severe sepsis or septic shock in the U.S. in 2012 was associated with interhospital transfer. While the exact indication for such transfer is unknown, the higher use of invasive medical devices and specialized medical technology such as ECMO and CRRT suggested these children were severely ill and in need of escalated care, likely prompting transfer.

Interhospital transfer is often indicative of a mismatch between a patient’s needs and the locally-available resources [14, 15], with the hope of salutary outcomes if such transfer was performed in a timely manner. Death from pediatric sepsis is often associated with multiple organ dysfunction the progression of which could be halted or reversed by timely institution of organ-supportive therapies [7, 16].

The study findings highlight significant variation in illness burden and resource-utilization by transfer status, with transfer hospitalizations more likely to involve children with extreme illness severity who were most often admitted to urban teaching hospitals. It was therefore not surprising that children who were transferred were more likely to undergo invasive medical device placement including arterial and central venous catheterization, tracheostomy, and invasive mechanical ventilation. This observation corroborates prior findings of higher resource utilization among critically ill patients who underwent inter-hospital transfer compared to those who did not [17]. Highly specialized and expensive technologies, including ECMO and CRRT, were similarly more likely to be deployed in the care of transferred children in comparison with non-transferred children. This finding of higher frequency of use of specialized technology among transferred versus non-transferred children, is likely indicative of greater illness severity among transferred children, corroborating prior report of an association between illness severity and deployment of invasive medical devices among the critically ill pediatric population [18]. The use of these technologies might have mitigated the impact of multiple organ dysfunction on patient death by limiting progression of organ dysfunction or enhancing resolution of organ dysfunction. Such assertion is supported by the observation that despite higher unadjusted mortality among transfer versus non-transfer hospitalizations, the mortality difference was no longer observed after multivariate adjustment for potential confounders including illness severity. This finding of similar severity-adjusted mortality also indicates the need to investigate processes of care and quality of care factors within the receiving urban hospitals that contributed to reduction in observed mortality.

Receiving hospitals bore a significant resource use burden reflected in their greater use of invasive medical technology along with the longer hospitalization and higher charges among transferred children in comparison to the non-transferred children. This disparate resource use burden was no longer evident after multivariate adjustment for potential factors that could confound the relationship between transfer status and resource use. The findings have implications for urban teaching hospitals that bear the brunt of these transfer hospitalizations. The first implication stems from the observation that higher charges and longer hospitalization among the transfer hospitalizations appeared commensurate to the severity of patient illness. Efforts to benchmark outcomes and resource use between hospitals that take care of critically ill children need to incorporate transfer admissions into algorithms for such inter-institutional assessments to avoid penalizing the receiving hospitals, as previously advocated for critically ill adults [19, 20]. Secondly, as an extension of this prior concept, the study findings highlight the need to routinely adjust in-hospital outcomes and resource use for illness severity and case mix [21, 22], without which hospitals that receive critically ill patients on interhospital transfer might be penalized for higher crude mortality and greater resource use.

The primary payer is an important variable that serves as a proxy for socioeconomic status. Public insurance coverage was associated with transfer status, a finding that corroborates recent work that noted significant variation in patient care processes, including transfer, by patients’ insurance coverage [23]. Given that such variation might impact patient outcomes [24], further work is warranted to investigate the reasons for such variation and the potential impact on outcomes and resource use in children with severe sepsis or septic shock.

The findings of the current study should be interpreted in light of certain limitations. It is vital to note that the reported hospital charges and LOS in the current study accrued from the care received at the hospital of definitive care thereby discounting the resource use expended at the referring hospital prior to interhospital transfer. As a result, the reported resource use burden accrued by the transferred children is likely an under-estimate of the actual burden. This limitation, imposed by the absence of linkable data for transferred children across hospitals in the data source for the study, highlights the need to conduct future longitudinal studies to provide a complete picture of the overall resource burden borne by children with pediatric sepsis who undergo transfer hospitalizations.

The KID is a database of administrative discharge data without clinical information beyond what can be captured in ICD-9-CM diagnosis and procedure codes. Therefore, it was not possible to study the clinical course for each patient, the need for and receipt of various therapeutic interventions, and how clinical care was coordinated at the patient and hospital level. An additional limitation is that the identification of cases of severe sepsis was ascertained via ICD-9-CM diagnosis codes, and is susceptible to inaccuracies of detection and attribution that may have biased our findings.

Also, while the care of patients with severe sepsis or septic shock might be improved by early resuscitation in the community prior to transfer to referral hospitals [7], determination of the characteristics of the referring hospitals and the circumstances surrounding transfer among the transfer hospitalizations, was not possible in the current study. This limited the ability to investigate any delay in resuscitative care, which might impact clinical outcomes and resource-utilization at the receiving hospital. Given the lack of clinical data, the characteristics and process of inter-hospital transport, and their potential impact on patient-level resource use and outcomes could not be ascertained. The KID contains non-clinical administrative data collected during patient hospitalizations and has no follow-up or longitudinal information on patients after hospital discharge. This limitation did not permit determination of long term patient morbidity and functional status.

## Conclusion

One in four children with severe sepsis or septic shock required interhospital transfer for specialized care associated with greater use of invasive medical devices and specialized technology. Despite higher crude mortality and resource consumption among transferred children, adjusted mortality and resource use did not differ by transfer status. Further research should identify quality-of-care factors at the receiving hospitals that influenced clinical outcomes and resource use for critically ill children with severe sepsis or septic shock.

## Data Availability

The KID is a publicly available dataset. The datasets used and analyzed during the current study are available from the corresponding author on reasonable request.

## Appendix A

### List of comorbidities [11]

Cardiovascular: Heart and great vessel malformations, cardiomyopathies, conduction disorders and dysrhythmias.

Respiratory: Respiratory malformations, chronic respiratory disease, and cystic fibrosis.

Renal and Urologic: Congenital anomalies, and chronic renal failure.

Neurologic and Neuromuscular: Brain and spinal cord malformations, mental retardation, central nervous system degeneration and disease, infantile cerebral palsy, epilepsy, muscular dystrophies and myopathies.

Gastrointestinal: Congenital anomalies, chronic liver disease and cirrhosis, inflammatory bowel disease.

Hematologic or immunologic: Sickle cell disease, hereditary anemias, hereditary immunodeficiency, human immunodeficiency virus disease.

Metabolic: Amino acid metabolism, carbohydrate metabolism, lipid metabolism, storage disorders; other metabolic disorders.

Other congenital or genetic defect: Chromosomal anomalies, bone and joint anomalies, diaphragm and abdominal wall, other congenital anomalies.

Malignancy

Neonatal

Technology dependency

Transplantation

## Appendix B

**Table 3:** Diagnosis codes used to identify organ dysfunction [12]

Organ Dysfunction	ICD-9-CM codes	Diagnosis
Respiratory	518.81	Acute respiratory failure
	518.82	Acute respiratory distress, insufficiency
	518.5	Pulmonary insufficiency following trauma and surgery
	799.1	Respiratory arrest, cardiorespiratory failure
	96.7	Mechanical ventilation
	518.84	Acute-on-chronic respiratory failure
Cardiovascular	785.50	Shock unspecified, failure of peripheral circulation
	785.59	Other shock, Septic
	458	Hypotension
	427.5	Cardiac arrest
Neurologic	293	Transient organic psychosis
	348.1	Anoxic brain injury
	348.3	Acute encephalopathy
	780.01	Coma
	780.09	Altered consciousness, unspecified
Hepatic	570	Acute and subacute necrosis of liver
	573.4	Hepatic infarction
Hematologic	286.6	Defibrination syndrome, disseminated intravascular coagulation
	286.9	Other and unspecified coagulation defects
	287.4	Secondary thrombocytopenia
	287.5	Thrombocytopenia, unspecified
Renal	584	Acute renal failure
	586	Renal failure, unspecified
	788.5	Oliguria, anuria

## Abbreviations

APRDRG: All-patient refined diagnostic related group
KID: Kids’ Inpatient Database
LOS: Length of stay
